# Proteomic analysis of serum samples of paracoccidioidomycosis patients with severe pulmonary sequel

**DOI:** 10.1371/journal.pntd.0009714

**Published:** 2021-08-23

**Authors:** Amanda Ribeiro dos Santos, Aline Dionizio, Mileni da Silva Fernandes, Marília Afonso Rabelo Buzalaf, Beatriz Pereira, Débora de Fátima Almeida Donanzam, Sergio Marrone Ribeiro, Anamaria Mello Miranda Paniago, Ricardo de Souza Cavalcante, Rinaldo Poncio Mendes, James Venturini

**Affiliations:** 1 Faculdade de Medicina, Universidade Federal de Mato Grosso do Sul (UFMS), Campo Grande, Brazil; 2 Faculdade de Medicina, Universidade Estadual Paulista (UNESP), Botucatu, Brazil; 3 Faculdade de Odontologia de Bauru (FOB), Universidade de São Paulo (USP), Bauru, Brazil; Universidad de Antioquia, COLOMBIA

## Abstract

**Background:**

Pulmonary sequelae (PS) in patients with chronic paracoccidioidomycosis (PCM) typically include pulmonary fibrosis and emphysema. Knowledge of the molecular pathways involved in PS of PCM is required for treatment and biomarker identification.

**Methodology/Principal findings:**

This non-concurrent cohort study included 29 patients with pulmonary PCM that were followed before and after treatment. From this group, 17 patients evolved to mild/ moderate PS and 12 evolved severe PS. Sera from patients were evaluated before treatment and at clinical cure, serological cure, and apparent cure. A nanoACQUITY UPLC-Xevo QT MS system and PLGS software were used to identify serum differentially expressed proteins, data are available via ProteomeXchange with identifier PXD026906. Serum differentially expressed proteins were then categorized using Cytoscape software and the Reactome pathway database. Seventy-two differentially expressed serum proteins were identified in patients with severe PS compared with patients with mild/moderate PS. Most proteins altered in severe PS were involved in wound healing, inflammatory response, and oxygen transport pathways. Before treatment and at clinical cure, signaling proteins participating in wound healing, complement cascade, cholesterol transport and retinoid metabolism pathways were downregulated in patients with severe PS, whereas signaling proteins in gluconeogenesis and gas exchange pathways were upregulated. At serological cure, the pattern of protein expression reversed. At apparent cure pathways related with tissue repair (fibrosis) became downregulated, and pathway related oxygen transport became upregulated. Additionally, we identified 15 proteins as candidate biomarkers for severe PS.

**Conclusions/Significance:**

Development of severe PS is related to increased expression of proteins involved in glycolytic pathway and oxygen exchange), indicative of the greater cellular activity and replication associated with early dysregulation of wound healing and aberrant tissue repair. Our findings provide new targets to study mechanisms of PS in PCM, as well as potential biomarkers.

## Author summary

Pulmonary fibrosis is the main sequel of paracoccidioidomycosis (PCM), a fungal disease that affects mainly men, rural workers. The development of pulmonary fibrosis is complex and involves several mechanisms that culminate in aberrant collagen production and deposition in the lungs making it became stiff and blocking the air passages. These changes lead to difficulty in breathing and in PCM patients dyspnea in response to high or low levels of exertion is common. Therefore, these patients show incapacity to work and the decreased quality of life. With the possibility of identifying some marker, for example, it could help the indication of respiratory physiotherapy, professional rehabilitation, or therapeutic intervention. This is the first study to examine the pulmonary sequelae (PS) in patients with paracoccidioidomycosis using an approach combining proteomics with bioinformatics. Here, we identify the specific proteome pattern found in PCM patients with severe sequelae that distinguishes these patients from that with mild/moderate sequelae. Our results showed that time points immediately before treatment and at clinical cure are key moments at which PS can progress to severe PS due a dysregulation in wound healing with consequent delayed in the healing processes resulting in an aberrant scar. As such, we suggest that the prognoses for severe PS should be considered as soon as possible and as early as diagnosis of PCM. Furthermore, we used proteomics to identify possible serum biomarkers with which to predict the likely development of severe PS, to be validated in future studies.

## Introduction

Paracoccidioidomycosis (PCM) is a granulomatous systemic mycosis caused by thermo-dimorphic fungi of the genus *Paracoccidioides* [1]. It is an endemic disease in Latin America and the primary cause of mortality among all endemic systemic mycoses in Brazil [2]. Clinical manifestations range from benign and localized to severe and disseminated [3]. The two main clinical presentations are the acute/subacute form (AF) and the chronic form (CF). CF PCM is the most common form of PCM; this form is usually observed in adult males, with clinical manifestations predominantly in the lungs and upper aerodigestive tract [4]. Even after appropriate antifungal treatment, most patients with CF present pulmonary sequelae (PS), including pulmonary fibrosis and emphysema [4, 5]. Patients with PS show incapacitating respiratory disorders that prevent them from performing previous occupational activities [6], and in some cases, this condition can trigger psychological problems and intensify the consumption of alcohol [7]. Despite representing a public health problem, PCM is a neglected disease [2, 3, 8].

Chronic inflammation associated with dysregulated wound healing results in fibrosis [9]. In the lung, this process is characterized by hyperplasia of myofibroblasts and intense deposition of collagens in the wall of the bronchial tree, blood vessels, and pulmonary parenchyma. These structural changes lead to a decline in lung function, which may be progressive [10]. Emphysema involves dramatic obliteration of the pulmonary alveoli as a result of an immunological response that leads to recruitment of inflammatory neutrophils, macrophages, and lymphocytes. These cells secrete matrix metalloproteinases, elastase, and other proteases, which destroy the alveolar wall [11].

In general, our understanding of the mechanisms underlying fibrosis and emphysema is based on studies performed in non-infectious pulmonary diseases [12, 13] such as asthma [14–16], chronic obstructive pulmonary disease (COPD)[17–19], and idiopathic pulmonary fibrosis [20–22]. These studies have clarified fibrogenesis mechanisms and identified biomarkers for prognostication and targeting of new drugs [23–29]. The clinical aspects of PCM PS have rarely been studied [5, 30], and although fibrogenesis in PCM is recognized as an early process [31–34], the molecular mechanisms involved in these sequela are still unknown. Our group has observed several immunological alterations in these patients even after successful antifungal treatment, including increased levels of pro-inflammatory cytokines and growth factor [35], high counts of CD14^+^CD16^++^ monocyte subsets [34], high counts of peripheral blood TCD4^+^ [35], increased counts of peripheral blood plasmacytoid dendritic cells [36], and enhanced inflammasome activation [37]. These findings highlights the complexity of a sequelae in these patients along with systemic repercussion.

Thus, the identification of molecular mechanisms for fibrogenesis would be of great value and can be found easily accessible on biological fluids. In addition, blood serum is one of the easiest accessible sources of biomarkers and its proteome presents a significant parcel of metabolism and immune system proteins, due this, the serum proteome analysis can provide not only biomarkers but also biological explanations for observed events [38]. Therefore, we hypothesized that patients with severe PS present different serum proteomic signature, before, during, and after antifungal treatment could help the identification of prognostic markers, molecular mechanisms, and therapeutic targets.

In the present study, we performed the first molecular study of PS in patients with PCM. Using a proteomic approach, we aimed to identify molecules involved in these sequelae by comparing groups of patients with severe and mild/moderate-intensity PS, and determine the significant signaling pathways at various stages of clinical follow-up. In addition, we aimed to identify serum proteins that could function as candidate predictive biomarkers of PS.

## Methods

### Ethics statement

All study participants gave their written informed consent for inclusion before they participated in the study. This study was conducted in accordance with the Brazilian Norms and Guidelines Research Regulators Involving Human Beings–(Res. CNS 196/96, II.4), International Guidelines for Biomedical Research involving Beings Human Rights–(CIOMS) and the principles of the Declaration of Helsinki. Ethical approval was obtained from the Ethical Committee of Botucatu Medical School, São Paulo State University, Brazil (CAAE: 65525317.9.3001.5398) on May 4, 2017.

### Study design and participants

This was a non-concurrent cohort study performed at the Tropical Diseases Ward and Systemic Mycoses Outpatient Clinic of the University Hospital (Botucatu Medical School, São Paulo State University) from 1995 to 2017. The samples were stored at -80 from a BioBank of Laboratory of Infectious Diseases(Botucatu Medical School, São Paulo State University). All patients presented with CF PCM and PS. PCM was confirmed by the presence of a suggestive clinical condition, and identification of the typical *P*. *brasiliensis* yeast form in one or more clinical materials and/or specific serum antibodies detected by a double immunodiffusion (DID) test at the stage of active disease. Inclusion criteria were PCM pulmonary involvement, blood collection at all four time points of antifungal treatment, and a chest computed tomography (CT) scan at the end of treatment. Exclusion criteria were the presence of other systemic diseases related to infection, inflammation, or neoplasia, pregnancy, and lactation.

### Procedures

We determined the serum proteome signature of 29 male patients, of whom 12 presented severe PS and 17 presented mild/moderate PS. There were no differences between the two groups in median age, degree of PCM severity, affected organs, type and time of treatment, time to clinical or serological cure, or specific serum antibody titer at admission (Table 1). Degree of PS was evaluated by CT scan after discontinuation of antifungal treatment. The analyzes of CT scan were performed by a team of experts and the classification was based on the types of lesions frequently associated with less or more extensive pulmonary damage. The presence of fibrotic nodules, bronchial wall thickening and small centrilobular nodules, or signs of focal paracicatricial emphysema with fibroatelectasis and discrete traction bronchiectasis around the foci, were considered signs of mild/moderate PS. More pronounced alterations, with extensive bronchiectasis associated with more extensive emphysema beyond the areas of fibrosis, honeycombing signs, hyper-transparent areas associated with vascular poverty, emphysema blisters, recessed and rectified domes, and pulmonary hyperinflation, were considered signs of severe PS.

**Table 1:** Clinical aspects of paracoccidioidomycosis patients distributed according of pulmonar sequel (PS) intensity. N (%).

Clinical Aspects		Patients (n = 29)	Mild/moderate PS (n = 17)	Severe PS (n = 12)	*p value*
**Age (years)** ^**1**^		50	56	57	*0*.*8003*
**Levels of severity** ^**2**^	**Mild**	2 (6.9)	2 (11.7)	0 (0)	*1*.*000*
	**Moderate**	23 (79.3)	13 (76.4)	10 (83.3)	
	**Severe**	4 (13.8)	2 (11.7)	2 (16.6)	
**Affected organs** ^**2**^	**Adrenal**	3 (10.3)	1 (5.88)	2 (16.6)	*0*.*367*
	**CNS**	3 (10.3)	3 (17.6)	0 (0)	*0*.*186*
	**Mucosa**	5 (51.7)	9(52.9)	6 (50)	*0*.*586*
	**Skin**	1 (3.4)	1 (5.88)	0 (0)	*0*.*586*
	**Lung**	29 (100)	17 (100)	12 (100)	*UR*
	**Larynx**	5 (17.2)	2 (11.7)	3(25)	*0*.*329*
	**Lymphnode**	4 (13.8)	4 (23.5)	0 (0)	*0*.*100*
	**Bone**	1 (3.4)	0 (0)	1 (8.33)	*0*.*413*
**Antifungal treatment** ^**2**^	**CMX**	17 (58.6)	11 (64.7)	6 (50)	*0*.*5368*
	**CMX–ITC**	4 (13.8)	4 (23.5)	0 (0)	
	**ITC**	3 (10.3)	1 (5.88)	2 (16.6)	
	**ITC- CMX**	2 (6.9)	0 (0)	2 (16.6)	
	**Others**	3 (10.3)	2 (11.7)	1 (8.3)	
**Time of treatment (months)** ^**1**^		38	28	44	*0*.*2871*
**Time for clinical cure (days)** ^**1**^		142	149	126	*0*.*2687*
**Time for serological cure (days)** ^**1**^ ^.^ ^**3**^		530	314.5	825	*0*.*2679*
**DID** ^**1**^		1:16	1:16	1:8	*0*.*9463*

All informations are expressed by median

^1^Mann-Whitney test

^2^ Fisher test

^3^ Kaplan-Meier curve

PS = Pulmonary Sequel; CNS = Central Nervous System UR = unrealized; CMX = cotrimoxazole; ITC = itraconazole; DID = double agar gel immunodifusion test

Patients from both groups were evaluated at four stages during follow-up, as described by Mendes et al. [3]; S0: before antifungal treatment; S1: clinical cure, characterized by the disappearance of the initial symptomatology, reversion of the erythrocyte sedimentation rate (ESR) to normal values, serological serum levels (as determined by DID tests) that are decreasing but usually positive, and ongoing antifungal treatment; S2: serological cure, characterized by clinical cure, a normal ESR, persistent negative DID serology for one year, and ongoing antifungal treatment; S3: apparent cure, characterized by clinical cure, a normal ESR, and a persistently negative DID for two years after the discontinuation of the treatment.

### Serum proteomics

Serum was collected after centrifugation of peripheral blood at 300 × *g* for 15 min, aliquoted, frozen at –80°C, and thawed once before proteomic analysis. Twelve patients with severe PS (with sera from four patients in each of three pools formed randomly and 17 patients with mild/moderate PS (with sera from five or six patients in each of three pools) were included in the study. Serum albumin and immunoglobulins were depleted using a ProteoPrep Blue Albumin and IgG Depletion Kit (Sigma-Aldrich, St Louis, MO, USA) according to manufacturer’s instructions. The expected depletion of these proteins was 80–95%. After depletion, Bradford assays [39] were performed to quantify proteins present in the pooled samples (n = 3 / group) and all samples were standardized to a concentration of 1μg/μL. Samples were submitted to proteomic analysis as previously described [40]. To 50 μL sample, 10 μL of 50 mM ammonium bicarbonate was added, before the following steps. First, 25 μL of 0.2% RapiGest (Waters Co., Manchester, UK) was added and incubated at 80°C for 15 min. Second, 2.5 μL of 100 mM dithiothreitol was added and incubated at 60°C for 30 min. Third, 2.5 μL of 300 mM iodoacetamide was added and incubated for 30 min at room temperature (in the dark). Fourth, 10 μL of trypsin (100 ng; Trypsin Gold, Mass Spectrometry Grade; Promega, Madison, WI, USA) was added and digestion was allowed to occur for 14 h at 37°C. Fifth, after digestion, 10 μl of 5% trichloroacetic acid was added, and the sample was left in an incubation phase for 90 min at 37°C. The sample was then centrifuged (16,000 g for 30 min). Finally, the supernatant was collected, and 5 μL of alcohol dehydrogenase (1 pmol/μL) plus 85 μL of 3% acetonitrile was added.

### LC-MS/MS and bioinformatics analyses

The NanoACQUITY UPLC-Xevo QT MS system (Waters Co., Manchester, UK) was used to separate and identify peptides using the ion count algorithm, exactly as previously described [41]. The software ProteinLynx GlobalServer (PLGS) version 3.0 (Waters, Milford, MA) was used to search the LC-MS continuum data. The identification of serum proteins was performed using Homo sapiens database from the UniProtKB (http://www.uniprot.org) in February 2018. All sample pools were analyzed in triplicate. To determine differences serum proteins expression between PCM patients with severe and mild/moderate PS in each stage of clinical follow-up, ProteinLynx Global Server (PLGS) Expression E software was used, with p < 0.05 and p > 0.95 used to identify downregulation and upregulation of proteins, respectively, as reported earlier [40]. The mass spectrometry proteomics data have been deposited to the ProteomeXchange Consortium via the PRIDE [42] partner repository with the dataset identifier PXD026906. Bioinformatics analysis was performed to compare groups, and UniProt protein ID accession numbers were mapped back to their associated encoding UniProt gene entries. Furthermore, the Reactome database of pathways was searched using ClueGo v2.0.7 + CluePedia v1.0.8, a Cytoscape plug-in. UniProt IDs were uploaded separately from Tables 1, 2, 3 and 4 and analyzed with default parameters, which specify an enrichment (right-sided hypergeometric test) statistical test with a Bonferroni step-down correction method, ‘single cluster’ analysis type, using the genecluster list for *Homo sapiens*, evidence codes ‘All’, networking specificity: medium (GO levels 3 to 8), and a kappa score threshold of 0.4. Due to the type of sample used in this study, many immunoglobulins were identified as proteins differentially expressed between the groups. Some abundant proteins such as immunoglobulins are known to mask other protein components that are present in low concentrations [43, 44]. We were unable to deplete more than IgG and albumin before the proteomic analysis; therefore, we decided not to include immunoglobulins in the tables and bioinformatic analysis because these were the predominant proteins with altered expression levels. However, these are listed in a supplementary tables (S1–S4 Tables).

**Table 2:** Proteins with expression significantly altered in the serum of paracoccidioidomycosis patients with severe and mild/moderate pulmonary sequel (PS) as outcome in the moment of before treatment (S0).

^a^Access number	Protein name	PLGS Score	^b^*Ratio* (severe PS:mild/moderate PS)
P68871	Hemoglobin subunit beta	5774	2.34
P69892	Hemoglobin subunit gamma-2	2216	2.32
P02042	Hemoglobin subunit delta	2216	2.29
P02100	Hemoglobin subunit epsilon	2216	2.27
P69891	Hemoglobin subunit gamma-1	2216	2.25
P69905	Hemoglobin subunit alpha	4673	2.16
P06733	Alpha-enolase	556	1.30
P09104	Gamma-enolase	556	1.30
P13929	Beta-enolase	556	1.30
P02766	Transthyretin	3869	1.17
P02750	Alpha-2-HS-glycoprotein	1470	0.88
P00739	Apolipoprotein A-I	5802	0.87
P00738	Serotransferrin	52105	0.82
P01023	Kininogen-1	436	0.80
P20742	Prothrombin	362	0.80
P01011	Inter-alpha-trypsin inhibitor heavy chain H4	94	0.79
P02749	Inter-alpha-trypsin inhibitor heavy chain H2	769	0.76
P01009	Clusterin	762	0.72
P0C0L4	Inter-alpha-trypsin inhibitor heavy chain H1	228	0.72
P0C0L5	Heparin cofactor 2	96	0.68
P02760	Vitronectin	455	0.67
P04217	Complement factor H	299	0.66
P01024	Vitamin D-binding protein	1196	0.64
P02790	Putative hydroxypyruvate isomerase	163	0.63
P00747	Ceruloplasmin	603	0.61
P00450	Complement factor B	1014	0.61
P00751	Hemopexin	3711	0.60
Q5T013	Plasminogen	238	0.60
P02774	Complement C3	9892	0.59
P08603	Alpha-1B-glycoprotein	995	0.58
P04004	Protein AMBP	607	0.53
P05546	Complement C4-B	316	0.52
P10909	Alpha-1-antitrypsin	3334	0.51
P19827	Complement C4-A	316	0.51
P19823	Beta-2-glycoprotein 1	847	0.51
Q14624	Alpha-1-antichymotrypsin	1432	0.50
P01042	Pregnancy zone protein	196	0.47
P00734	Alpha-2-macroglobulin	4747	0.44
P02787	Haptoglobin	19050	0.43
P02647	Haptoglobin-related protein	8443	0.43
P02765	Leucine-rich alpha-2-glycoprotein	124	0.36
*Q6DKI1	60S ribosomal protein L7-like 1	34	Severe PS*
P01019	Angiotensinogen	109	Severe PS
P01031	Complement C5	32	Severe PS
Q9H6N6	Putative uncharacterized protein MYH16	49	Severe PS
Q6ZNX1	Shieldin complex subunit 3	36	Severe PS
*P02763	Alpha-1-acid glycoprotein 1	493	mild/moderate PS
*P19652	Alpha-1-acid glycoprotein 2	1020	mild/moderate PS
*Q02985	Complement factor H-related protein 3	20	mild/moderate PS
*Q5SZK8	FRAS1-related extracellular matrix protein 2	12	mild/moderate PS
*P17301	Integrin alpha-2	37	mild/moderate PS
*O60229	Kalirin	37	mild/moderate PS
*Q86W24	NACHT_ LRR and PYD domains-containing protein 14	16	mild/moderate PS
*P05155	Plasma protease C1 inhibitor	192	mild/moderate PS
*O94827	Pleckstrin homology domain-containing family G member 5	72	mild/moderate PS
*Q9NZ71	Regulator of telomere elongation helicase 1	70	mild/moderate PS
*P02753	Retinol-binding protein 4	104	mild/moderate PS

^**a**^ Identification is based on proteins ID from UniProt protein database, reviewed only (http://www.uniprot.org).

^**b**^ Proteins with expression significantly altered are organizaed according to the ratio.

* Indicates unique proteins in alphabetical order.

**Table 3:** Proteins with expression significantly altered in the serum of paracoccidioidomycosis patients with severe and mild/moderate pulmonary sequel (PS) as outcome in the moment of clinical cure (S1).

^a^Access number	Protein name	PLGS Score	^b^*Ratio* (severe PS:mild/moderate PS)
P69905	Hemoglobin subunit alpha	2896	1.72
P02787	Serotransferrin	44194	1.30
P00738	Haptoglobin	21516	1.16
P00739	Haptoglobin-related protein	10216	1.14
P68871	Hemoglobin subunit beta	4951	1.11
P04217	Alpha-1B-glycoprotein	948	0.93
P01011	Alpha-1-antichymotrypsin	2832	0.90
P08603	Complement factor H	284	0.88
P19827	Inter-alpha-trypsin inhibitor heavy chain H1	328	0.87
P02774	Vitamin D-binding protein	2264	0.87
P02751	Fibronectin	94	0.86
P00747	Plasminogen	308	0.85
P02749	Beta-2-glycoprotein 1	905	0.84
P0C0L4	Complement C4-A	470	0.84
P0C0L5	Complement C4-B	470	0.84
P05546	Heparin cofactor 2	94	0.84
P04004	Vitronectin	763	0.82
P01024	Complement C3	11714	0.81
P19823	Inter-alpha-trypsin inhibitor heavy chain H2	868	0.79
P01023	Alpha-2-macroglobulin	8579	0.79
P20742	Pregnancy zone protein	378	0.79
P00450	Ceruloplasmin	1877	0.78
P01042	Kininogen-1	320	0.77
P02760	Protein AMBP	590	0.76
P01008	Antithrombin-III	145	0.75
P02790	Hemopexin	4428	0.72
P01009	Alpha-1-antitrypsin	5216	0.70
P00734	Prothrombin	501	0.70
P10909	Clusterin	697	0.69
P06727	Apolipoprotein A-IV	131	0.65
P02766	Transthyretin	1202	0.62
P02647	Apolipoprotein A-I	4009	0.58
P02656	Apolipoprotein C-III	1698	0.36
P02763	Alpha-1-acid glycoprotein 1	157	Severe PS*
P01019	Angiotensinogen	272	Mild/moderate PS
P02649	Apolipoprotein E	130	Mild/moderate PS
P02753	Retinol-binding protein 4	437	Mild/moderate PS
P27169	Serum paraoxonase/arylesterase 1	1266	Mild/moderate PS

^a^ Identification is based on proteins ID from UniProt protein database. reviewed only (http://www.uniprot.org).

^**b**^ Proteins with expression significantly altered are organized according to the ratio.

* Indicates unique proteins in alphabetical order.

**Table 4:** Proteins with expression significantly altered in the serum of paracoccidioidomycosis patients with severe and mild/moderate pulmonary sequel (PS) as outcome in moment of serological cure (S2).

^a^Access number	Protein name	PLGS Score	^b^*Ratio* (severe PS:mild/moderate PS)
P02751	Fibronectin	799	2.32
P05155	Plasma protease C1 inhibitor	561	1.82
P00739	Haptoglobin-related protein	7979	1.39
P00738	Haptoglobin	20464	1.35
Q14624	Inter-alpha-trypsin inhibitor heavy chain H4	56	1.34
Q5T013	Putative hydroxypyruvate isomerase	140	1.32
P02787	Serotransferrin	58693	1.25
P02765	Alpha-2-HS-glycoprotein	1410	1.22
P02750	Leucine-rich alpha-2-glycoprotein	280	1.21
P13929	Beta-enolase	1050	1.19
P09104	Gamma-enolase	1050	1.19
P01024	Complement C3	21361	1.16
P00751	Complement factor B	1405	1.15
P02790	Hemopexin	5861	1.15
P04004	Vitronectin	693	1.15
P04196	Histidine-rich glycoprotein	110	1.15
P05546	Heparin cofactor 2	158	1.12
P00747	Plasminogen	465	1.09
P01011	Alpha-1-antichymotrypsin	2056	1.08
P02647	Apolipoprotein A-I	8940	1.07
P10909	Clusterin	1717	1.07
P20742	Pregnancy zone protein	301	1.06
P02774	Vitamin D-binding protein	2536	1.05
P00450	Ceruloplasmin	1149	1.04
P01023	Alpha-2-macroglobulin	9840	1.03
P06727	Apolipoprotein A-IV	126	0.91
P0C0L4	Complement C4-A	470	0.91
P0C0L5	Complement C4-B	460	0.90
P02760	Protein AMBP	336	0.89
P01009	Alpha-1-antitrypsin	6126	0.84
P02749	Beta-2-glycoprotein 1	1341	0.78
P02763	Alpha-1-acid glycoprotein 1	225	0.61
P27169	Serum paraoxonase/arylesterase 1	726	0.57
P69905	Hemoglobin subunit alpha	386	0.52
P19652	Alpha-1-acid glycoprotein 2	123	0.51
P68871	Hemoglobin subunit beta	2416	0.50
P02042	Hemoglobin subunit delta	1332	0.47
P02100	Hemoglobin subunit epsilon	1332	0.46
P69891	Hemoglobin subunit gamma-1	1332	0.46
P69892	Hemoglobin subunit gamma-2	1332	0.45
P08697	Alpha-2-antiplasmin	689	Severe PS*
P02652	Apolipoprotein A-II	436	Severe PS
P02649	Apolipoprotein E	158	Severe PS
P0DJI8	Serum amyloid A-1 protein	3449	Severe PS
P0DJI9	Serum amyloid A-2 protein	544	Severe PS
Q15166	Serum paraoxonase/lactonase 3	4	mild/moderate PS

^a^ Identification is based on proteins ID from UniProt protein database. reviewed only (http://www.uniprot.org).

^**b**^ Proteins with expression significantly altered are organized according to the ratio.

* Indicates unique proteins in alphabetical order.

### Dosage of serum mediators

The serum levels of SPD, MIP-1α, IL-10, TNF- α, IL-1 β, TGF- β, FGF, VEGF, PDGF were quantified by the DuoSet@ ELISA Development kit (R&D systems, Minneapolis, MN, USA).

### Statistical analysis

Clinical data were analyzed as follows. Homogeneity of patient groups was examined by the Chi-square or Fisher’s exact test. Mann-Whitney U test was used to analyze the time to serological and apparent cure. Unpaired t-test was used to compare two independent samples. Statistical analyses were performed using SAS Version 9.3 and GraphPad v.5.00 (GraphPad Software Inc., San Diego, CA, USA) software. Significance level was set at *p* ≤0.05. For proteomics data, statistical analysis followed the specifications of each algorithm [45] as previously described.

## Results

### Comprehensive global proteome profiling of serum proteins in severe PS patients compared with the serum of PCM patients with mild/moderate PS

**Fig 1:**
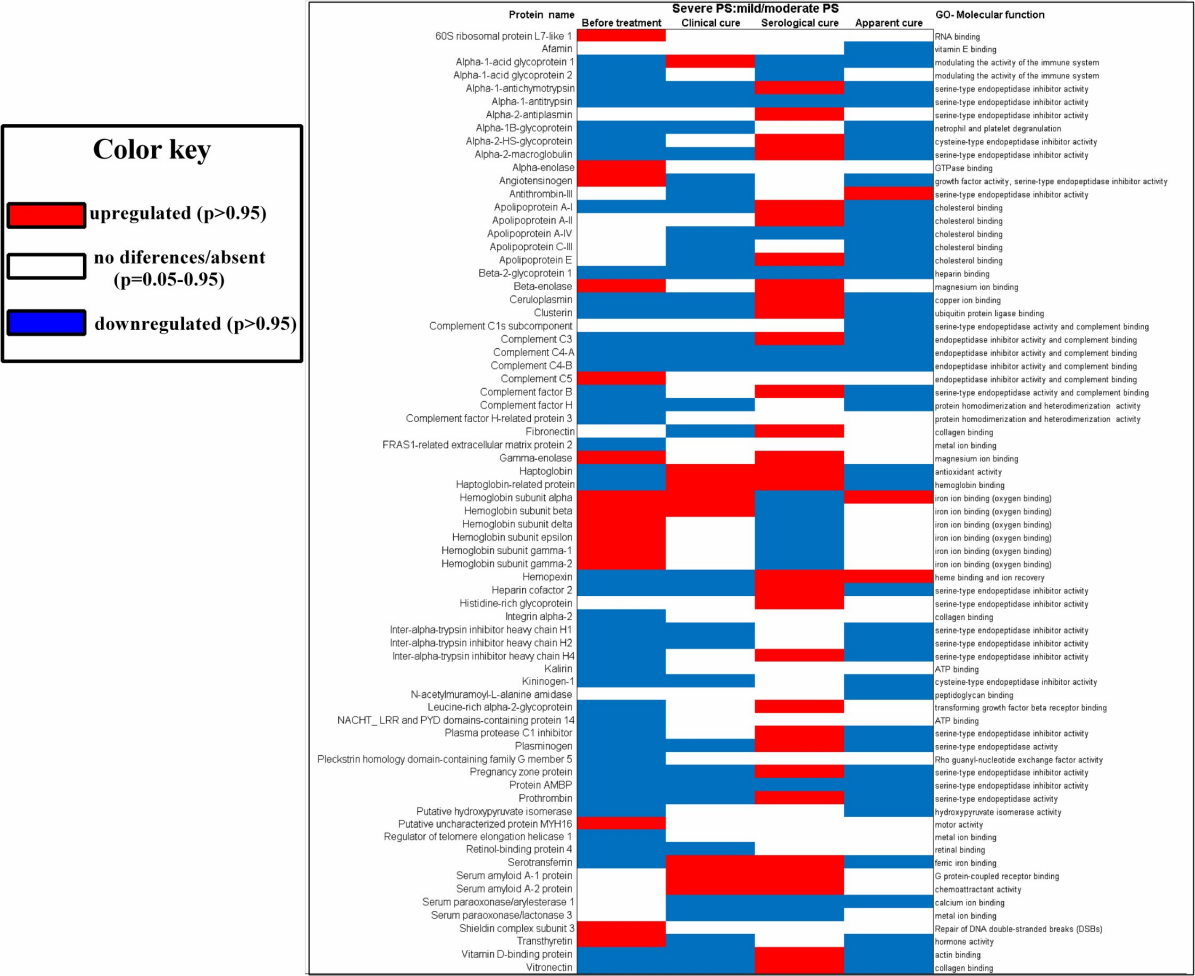
Heatmap of proteins differentially expressed in the sera of patients with severe pulmonary sequelae (PS) compared to patients with mild/moderate PS, at various stages during clinical follow-up. Upregulated proteins (p > 0.95) are shown in red, downregulated proteins (p < 0.05) are shown in blue, and proteins without significant differences between the groups are colorless. GO: gene ontology; PS: pulmonary sequelae.

During clinical follow-up, 72 proteins were identified as differentially expressed in the serum of PCM patients with severe PS compared with the serum of PCM patients with mild/moderate PS. The biological functions of these 72 proteins, according to the UniProt protein database, and their expression levels in patients with severe PS relative to those in patients with mild/moderate PS, are displayed in a heatmap (Fig 1).

### Reactome pathways analysis of serum proteins in severe PS patients, immediately before treatment (S0)

**Fig 2:**
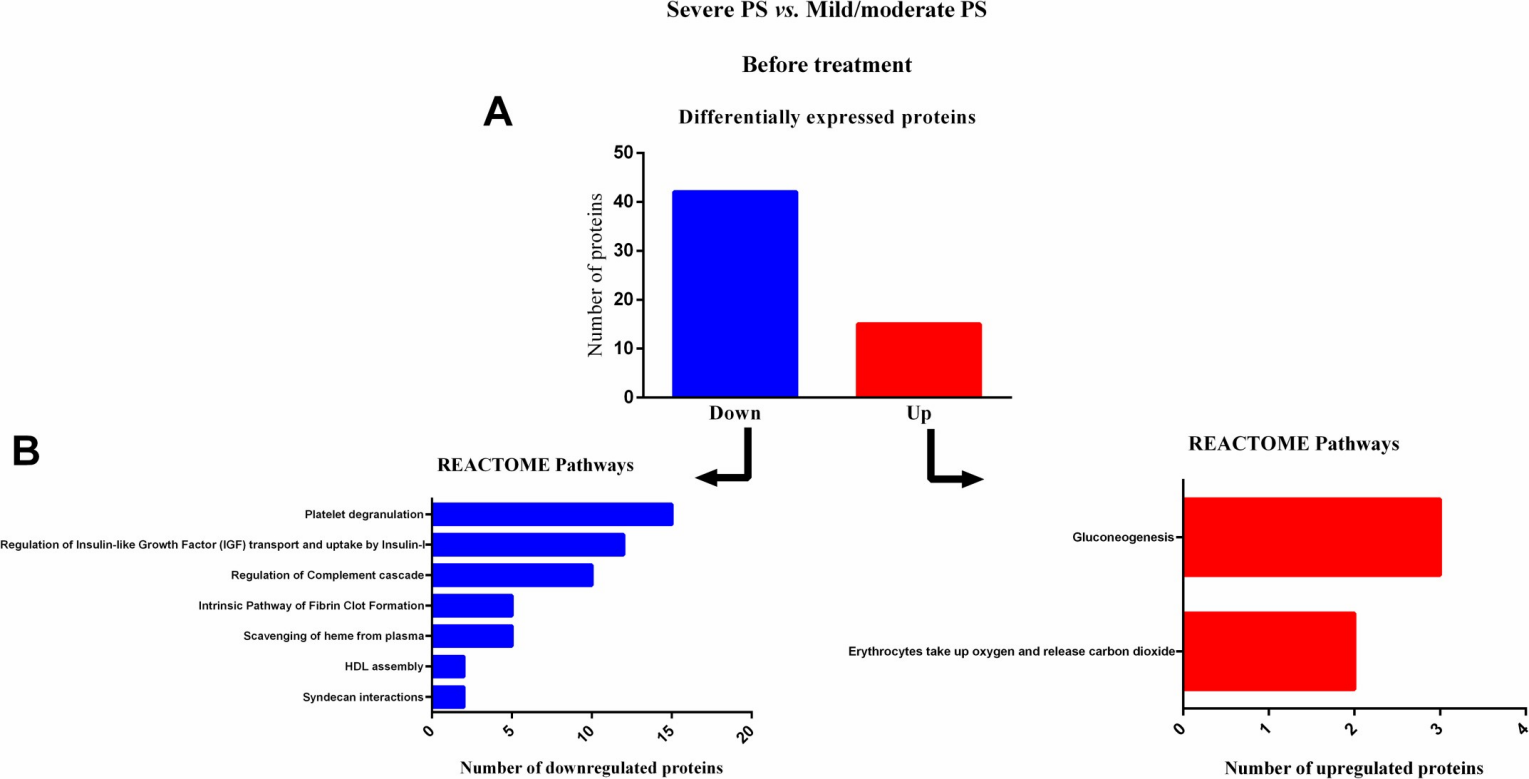
Enrichment analysis of serum proteins with significantly altered expression in paracoccidioidomycosis (PCM) patients with severe pulmonary sequelae (PS), compared to PCM patients with mild/moderate PS, immediately before treatment (S0). (A) Number of proteins down- or upregulated in PCM patients with severe PS compared to patients with mild/moderate PS. (B) Reactome pathway enrichment analysis of downregulated proteins (left) and upregulated proteins (right). All statistically significant pathways are listed. Significant terms (kappa = 0.4). HDL: high-density lipoprotein; PS: pulmonary sequelae.

Table 2 shows changes in protein expression in patients with severe PS compared to those with mild/moderate PS, before treatment (S0). Fig 2 shows that of 57 differentially expressed proteins, 42 were downregulated and 15 were upregulated in patients with severe PS compared to patients with mild/moderate PS (Fig 2A). Down- and upregulated proteins were separately uploaded into Cytoscape software to evaluate Reactome pathways. As can be observed in Fig 2B, the downregulated proteins were significantly enriched in seven different pathways, whilst the upregulated proteins were enriched in two different pathways.

### Reactome pathways analysis of serum proteins in severe PS patients, at clinical cure (S1)

**Fig 3:**
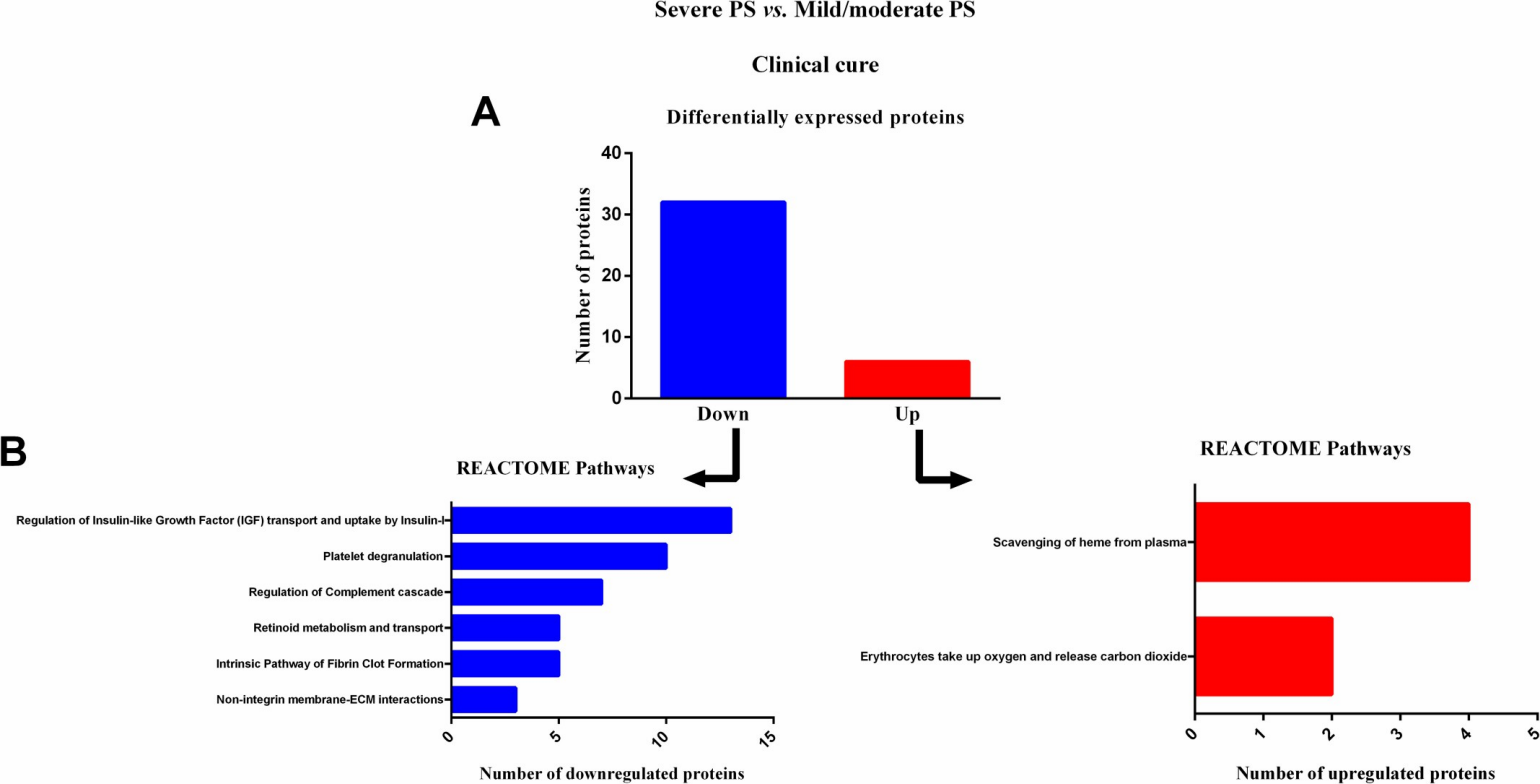
Enrichment analysis of serum proteins with significantly altered expression in paracoccidioidomycosis (PCM) patients with severe pulmonary sequelae (PS) compared to PCM patients with mild/moderate PS, at clinical cure. (A) Number of proteins down- or upregulated in PCM patients with severe PS compared to patients with mild/moderate PS. (B) Reactome pathway enrichment analysis of downregulated proteins (left) and upregulated proteins (right). All statistically significant pathways are listed. Significant terms (kappa = 0.4). ECM: extracellular matrix; PS: pulmonary sequelae.

At clinical cure (S1), 38 differentially expressed proteins were identified in patients with severe PS when compared to patients with mild/moderate PS (Table 3). Of these, 32 were downregulated and six were upregulated in patients with severe PS (Fig 3A). As observed in Fig 3B, Reactome pathway analysis revealed that most downregulated proteins were involved in six different pathways, while the upregulated proteins were involved in two pathways.

### Reactome pathways analysis of serum proteins in severe PS patients, at serological cure (S2)

**Fig 4:**
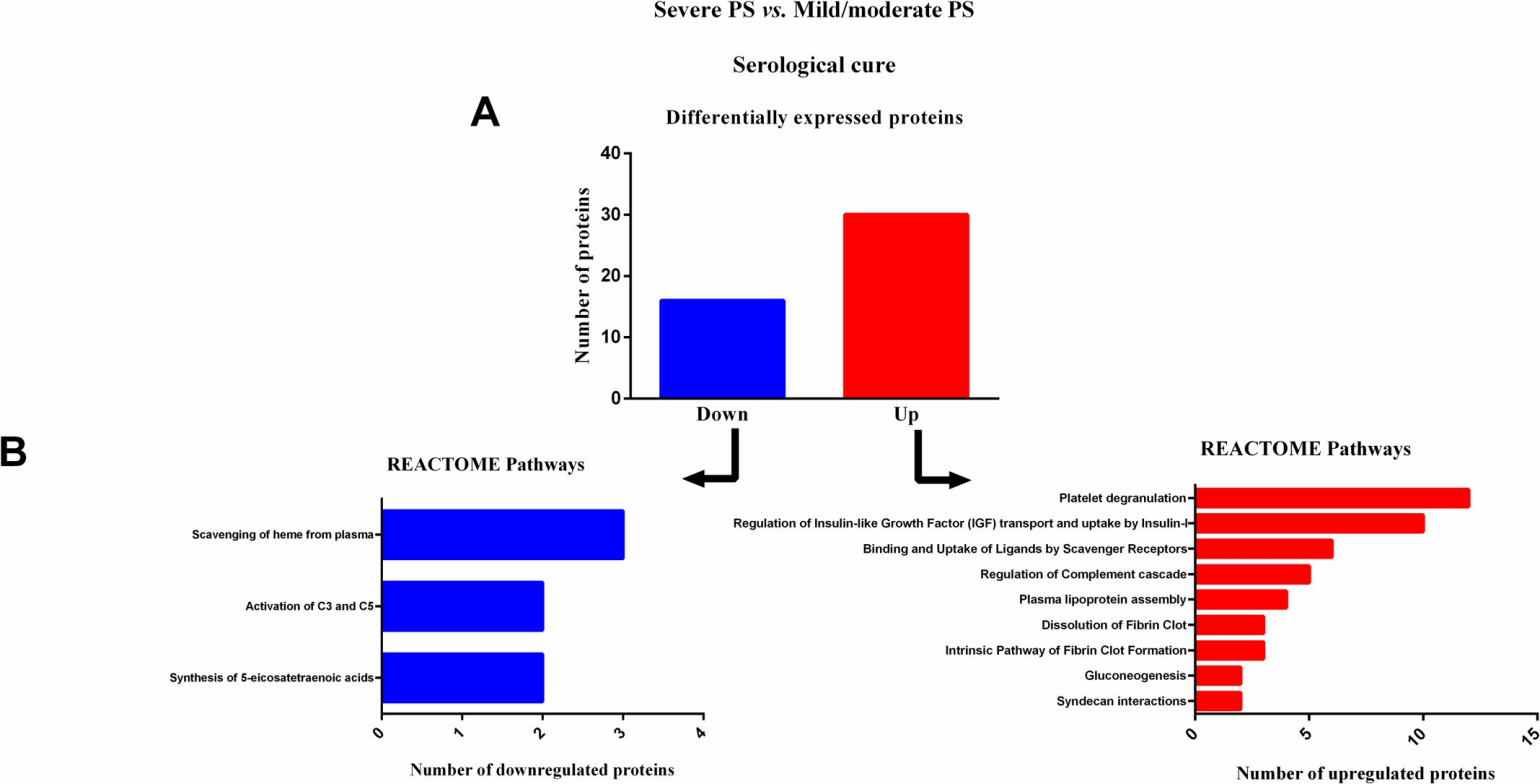
Enrichment analysis of serum proteins with significantly altered expression in paracoccidioidomycosis (PCM) patients with severe pulmonary sequelae (PS) compared to patients with mild/moderate PS, at serological cure (S2). (A) Number of proteins down- or upregulated in PCM patients with severe PS compared to patients with mild/moderate PS. (B) Reactome pathway enrichment analysis of downregulated proteins (left) and upregulated proteins (right). All statistically significant pathways are listed. Significant terms (kappa = 0.4). C3:complement C3; C4: complement C4; PS: pulmonary sequelae.

At serological cure (S2), patients with severe PS showed 46 differentially expressed proteins (Table 4) when compared with patients with mild/moderate PS, and of these, 16 were downregulated and 30 were upregulated (Fig 4A). Reactome pathway analysis revealed that most downregulated proteins were involved in three different pathways, whilst the upregulated proteins were involved in nine pathways (Fig 4B).

### Reactome pathways analysis of serum proteins in severe PS patients, at apparent cure (S3)

**Fig 5:**
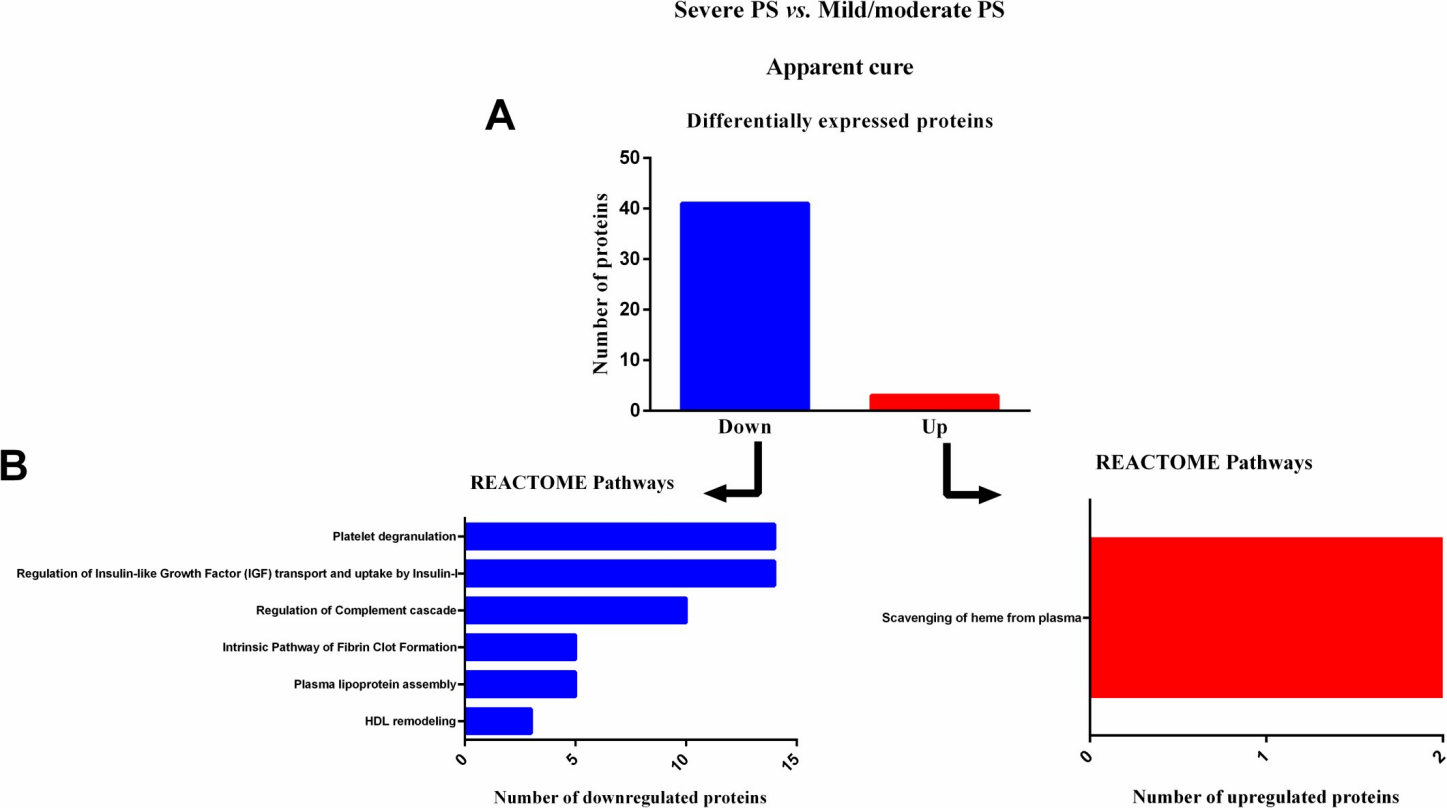
Enrichment analysis of serum proteins with significantly altered expression in paracoccidioidomycosis (PCM) patients with severe pulmonary sequelae (PS) compared to patients with mild/moderate PS, at apparent cure (S3). (A) Number of proteins down- or upregulated in PCM patients with severe PS compared to patients with mild/moderate PS. (B) Reactome pathway enrichment analysis of downregulated proteins (left) and upregulated proteins (right). All statistically significant pathways are listed. Significant terms (kappa = 0.4). HDL: high-density lipoprotein; PS: pulmonary sequelae.

Finally, at apparent cure (S3), 44 differentially expressed proteins were identified in patients with severe PS compared to patients with mild/moderate PS (Table 5). Of these, 41 were downregulated and three were upregulated (Fig 5A) in patients with severe PS. Reactome pathway analysis revealed that most downregulated proteins were involved in five six different pathways, and upregulated proteins were involved in one signaling pathway (Fig 5B).

**Table 5:** Proteins with expression significantly altered in the serum of Paracoccidioidomycosis patients with Severe and Mild/moderate pulmonary sequel (PS) as outcome in the moment of apparent cure.

^a^Access number	Protein name	PLGS Score	^b^*Ratio* (Severe PS:mild/moderate PS)
P69905	Hemoglobin subunit alpha	1823	1.30
P02790	Hemopexin	6961	1.06
P08603	Complement factor H	486	0.88
P00738	Haptoglobin	22790	0.87
P01023	Alpha-2-macroglobulin	6037	0.85
P00739	Haptoglobin-related protein	9541	0.84
P20742	Pregnancy zone protein	432	0.84
P00751	Complement factor B	1196	0.83
P02749	Beta-2-glycoprotein 1	1413	0.78
P02774	Vitamin D-binding protein	1889	0.77
P00747	Plasminogen	337	0.76
P04004	Vitronectin	705	0.76
Q96PD5	N-acetylmuramoyl-L-alanine amidase	144	0.74
P01042	Kininogen-1	732	0.74
P0C0L4	Complement C4-A	489	0.73
P0C0L5	Complement C4-B	488	0.73
P00734	Prothrombin	541	0.73
P05546	Heparin cofactor 2	93	0.72
P02766	Transthyretin	2160	0.72
P01011	Alpha-1-antichymotrypsin	1925	0.71
P04217	Alpha-1B-glycoprotein	999	0.68
P00450	Ceruloplasmin	2315	0.66
P01024	Complement C3	13144	0.66
P19827	Inter-alpha-trypsin inhibitor heavy chain H1	1036	0.66
P01019	Angiotensinogen	117	0.65
P02760	Protein AMBP	495	0.65
P02787	Serotransferrin	45136	0.65
Q14624	Inter-alpha-trypsin inhibitor heavy chain H4	147	0.64
P19823	Inter-alpha-trypsin inhibitor heavy chain H2	1325	0.63
Q5T013	Putative hydroxypyruvate isomerase	72	0.60
P02656	Apolipoprotein C-III	4030	0.59
P02765	Alpha-2-HS-glycoprotein	2620	0.58
P10909	Clusterin	823	0.58
P06727	Apolipoprotein A-IV	155	0.56
P01009	Alpha-1-antitrypsin	4246	0.55
P02647	Apolipoprotein A-I	8083	0.52
P01008	Antithrombin-III	156	Severe PS*
P43652	Afamin	120	mild/moderate PS
P02763	Alpha-1-acid glycoprotein 1	279	mild/moderate PS
P02652	Apolipoprotein A-II	547	mild/moderate PS
P02649	Apolipoprotein E	486	mild/moderate PS
P09871	Complement C1s subcomponent	65	mild/moderate PS
P05155	Plasma protease C1 inhibitor	90	mild/moderate PS
P27169	Serum paraoxonase/arylesterase 1	857	mild/moderate PS

^a^ Identification is based on proteins ID from UniProt protein database. reviewed only (http://www.uniprot.org).

^**b**^ Proteins with expression significantly altered are organized according to the ratio.

* Indicates unique proteins in alphabetical order.

### Overview of serum proteins differentially expressed between patients with severe PS, at various stages of clinical follow-up

**Fig 6:**
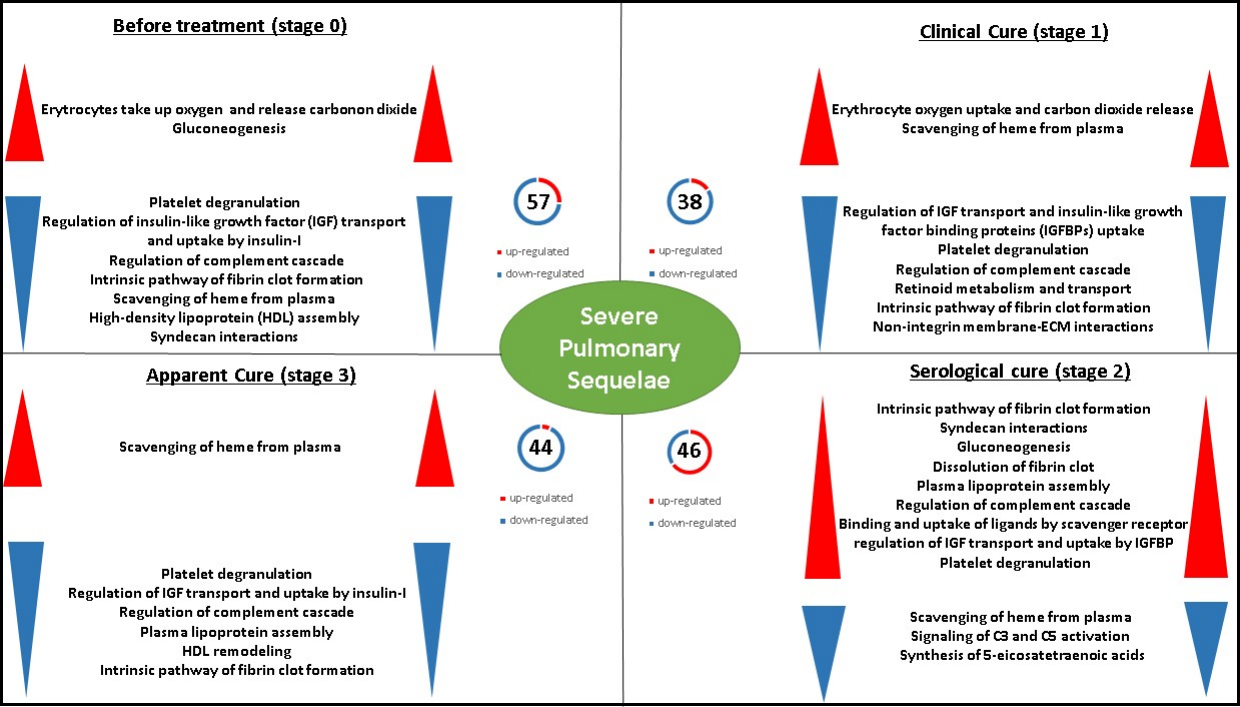
Overview of serum proteins differentially expressed between patients with chronic paracoccidioidomycosis developing severe pulmonary sequelae (PS) and those developing mild/moderate PS, at various stages of clinical follow-up. NanoACQUITY UPLC-Xevo QT MS system and PLGS Expression E software were used to identify differentially expressed proteins that were then categorized based on the Reactome pathway database. Significant terms (kappa = 0.4). HDL: high-density lipoprotein; ECM: extracellular matrix;. C3:complement C3; C4: complement C4; IGF: insulin-like growth factor; IGFBP: insulin-like growth factor binding protein.

Analysis of the pathways implicated across all PCM stages showed that the 72 differentially expressed proteins identified in this study were involved in 12 different pathways participating in pulmonary tissue wound healing (Fig 6). At S0, pathways related with the initial steps of wound healing were downregulated in patients with severe PS compared to patients with mild/moderate PS, while the ‘erythrocytes take up oxygen and release carbon dioxide’, and gluconeogenesis pathways were upregulated. The expression pattern of these pathways changed after the introduction of antifungals. The expression of proteins involved in these pathways reversed slowly; at serological cure (S2), an upregulation of the pathways related to the initial phases of wound healing was observed, whilst there was no change in the ‘erythrocytes take up oxygen and release carbon dioxide’ pathway. Conversely, at apparent cure (S3), alterations in these pathways were now observed in the opposite direction: pathways related with tissue repair (fibrosis) became downregulated, and pathways related with gas exchange processes, e.g. scavenging of heme from plasma, became upregulated.

### Serum biomarkers of inflammatory and fibrotic processes before treatment (S0)

**Fig 7:**
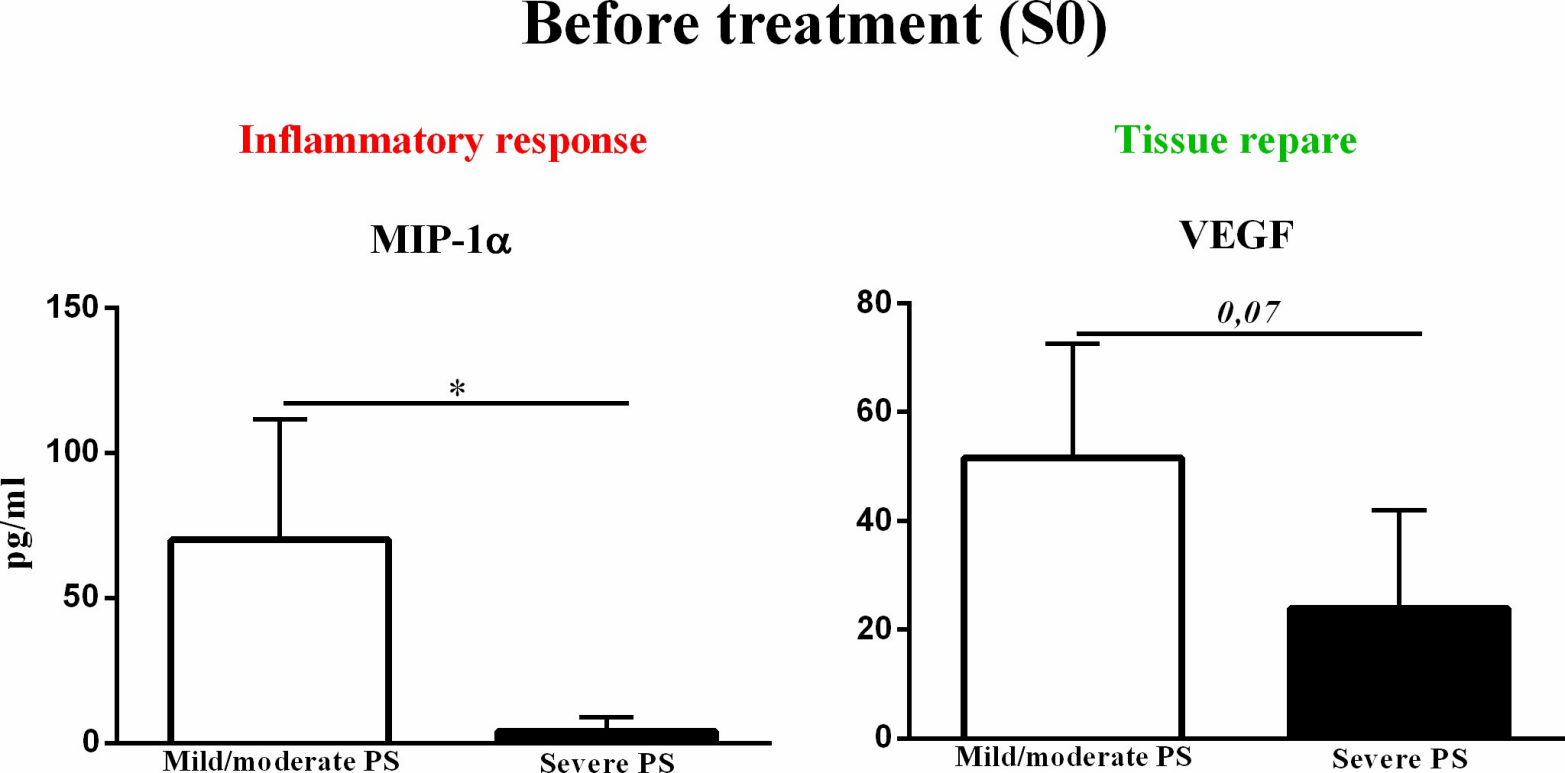
Serum biomarkers of inflammatory and fibrotic processes associated with mild/moderate or severe pulmonary sequelae (PS) outcomes in patients with chronic paracoccidioidomycosis. Serum concentrations of MIP-1α and VEGF in chronic PCM patients with mild/moderate or severe pulmonary sequelae as outcomes immediately before treatment (S0). Group differences were analyzed by unpaired t-test. MIP-1α: macrophage inflammatory protein 1 alpha; PCM: paracoccidioidomycosis; VEGF: vascular endothelial growth factor. PS: pulmonary sequelae.

In order to to validate the findings of proteomic data, serum levels of SPD, MIP-1α, IL-10, TNF- α, IL-1 β, TGF- β, FGF, VEGF, and PDGF were measured in chronic PCM patients with mild/moderate or severe PS. In accordance with our proteomic findings, we found that at moment before treatment (S0) serum concentration of MIP-1α and VEGF were lower and in patients with severe PS compared to patients with mild/moderate PS (Fig 7). No differences were found in serum levels of SPD, IL-10, TNF- α, IL-1 β, TGF- β, FGF, and PDGF between the two groups.

## Discussion

Wound healing is a dynamic and highly regulated process consisting of cellular, humoral, and molecular mechanisms [46], with many opportunities for dysregulation, and thus the potential to lead to numerous pulmonary disorders [47]. Most of the studies on pulmonary wound healing have been carried out in patients with inflammatory, non-infectious diseases. The present study was performed in patients with PCM, a chronic granulomatous infectious disease, classified into two groups by the severity of their pulmonary sequelae: mild/moderate, or severe. The serum proteome signature of these two groups were compared at different stages of the disease, from the active stage until the apparent cure.

In this study, 72 proteins were found to have altered expression across different PCM stages in patients with severe PS compared to patients with mild/moderate PS. These proteins were identified as participating in pathways important in the wound healing process. The physiological response to wounds can be characterized by key stages, including hemorrhage and fibrin-clot formation, inflammatory responses, re-epithelialization, granulation tissue formation, angiogenic responses, connective tissue contraction, and remodeling [48]. In healthy tissue, this process is likely to happen continuously at a background level to maintain homeostasis. However, in chronic lung disease, repair processes are not able to adequately offset the injurious process, and aberrant repair fails to restore normal epithelial integrity, leading to loss of lung function [47].

At S0 and S1, the pathways related to the initial steps of wound healing, including coagulation, such as fibrin clot formation [49–52] and platelet degranulation plugs [53]; pro-inflammatory responses including complement cascade and syndecan interactions [54, 55]; release of growth factors, including insulin-like growth factor [56–58]; and essential steps in the healing process, such as non-integrin membrane-extracellular matrix (ECM) interactions [59–61], were less active in patients with severe PS than in patients with mild/moderate PS. In addition, pathways involved in the protective tissue repair processes, including high-density lipoprotein assembly [62–64] and retinoid metabolism [65] pathways, were also downregulated in patients with severe PS. These data suggest lower wound healing activity in patients with severe PS than in patients with mild/moderate PS, in this active phase of PCM. In accordance with our proteomic findings, at S0, levels of a pro-inflammatory cytokine, macrophage inflammatory protein 1 alpha (MIP-1α), and a grown factor mediator, vascular endothelial growth factor (VEGF), were lower in patients with severe PS when compared with patients with mild/moderate, as measured by enzyme-linked immunosorbent assay. MIP-1α is a member of the C-C subfamily of chemokines, inducible proteins that exhibit various proinflammatory activities *in vitro* including leukocyte chemotaxis[66] whilst VEGF has an important role in wound healing through angiogenesis [67, 68]. Furthermore, it is known that a less effective *Paracoccidioides*-specific T-cell mediated response leads to a chronic infection, in which causes a dysregulated wound response, and inducing the development of pulmonary sequelae. The lower levels of these pro-inflammatory and growth factor mediators, allied to downregulation of wound healing pathways, in the sera of patients with severe PS indicate a dysregulation in tissue repair during the active phase of PCM.

On the other hand, at S0, the erythrocytes take up oxygen and release carbon dioxide, and enzymes alpha, gamma and beta enolases that are participating of gluconeogenesis and glycolytic pathways were upregulated in patients with severe PS compared with those with mild/moderate PS. It is well-known that during ECM production, fibroblasts have high glycolytic flux and biosynthetic activity even when they are not growing [69]. To prevent fibrosis, ECM anabolism and catabolism need to be aligned and tightly controlled. It was observed that a consistent downregulation in fatty acid oxidation and upregulation of glycolysis in fibrotic skin and in normal skin with abundant ECM [70]. In addition to the an increased glycolytic activity, the higher activity of erythrocytes take up oxygen, indicating increased levels of oxygen in wound healing process. In the chronic wound microenvironment, there inevitably exists a substantial imbalance between the supply of oxygen and the high energy demand of the healing tissue [71]. From a molecular standpoint, the key factors that propagate this imbalance include the following: (1) the increased utilization of oxygen by the hypermetabolic regenerating tissue, (2) the sustained and increased production of ROS by phagocytes (respiratory burst), and (3) reduction-oxidation (redox) signaling [71]. Furthermore, oxygen is needed in the later steps of collagen synthesis for proline and lysine hydroxylation and cross-linking, which is the step required for collagen to be released from cells [72]. The upregulation of these two pathways at the same time in severe PS patients when compared with mild/moderate PS patients in during the active phase of PCM indicating higher ECM deposition [59, 60, 69, 70, 73], which is related with the development of more fibrosis [47]. At the same way, an excess of ECM deposition is a hallmark of chronic progressive scarring conditions that fall under the fibrotic interstitial lung disease umbrella, including IPF [47].

Corroborating our findings, Tobón and collaborators [30], analyzing clinical records and chest radiographs from 47 itraconazole-treated patients with PCM undergoing prolonged post-therapy follow-up, found fibrotic lesions in 31.8% of patients at diagnosis (S0), and, at the end of the study, fibrosis persisted in these patients as sequelae. Unfortunately, the study did not classify sequelae severity at the end of follow-up. Taken together, our data suggest that patients who progressed to severe PS showed, at active disease (S0), more delayed wound healing responses than patients progressing to mild/moderate PS, characterized by lower activity of pathways important for tissue repair, and associated with overproduction of ECM, causing early fibrosis.

This profile persisted in the evaluation performed at S1, at which time patients were receiving antifungal treatment and had presented clinical cure. These findings indicate that the pathways responsible for the initial stages of tissue repair remain downregulated despite the clinical improvement followed by clinical cure. These pathways changed at the stage of serological cure (S2); the heatmap shown in Fig 1 illustrates an upregulation of the proteins involved in pathways related to wound healing [49–53, 55–58, 62–64]. Interestingly, the evaluation carried out at apparent cure (S3) showed a further alteration to these pathways, but in the opposite direction, i.e., the pathways related with tissue repair (fibrosis) were downregulated, and the pathway related to the scavenging of heme from plasma was upregulated, favoring the activities related to gas exchange [74]. As the pulmonary sequelae are also deleterious for patients due to its interference in respiratory function [5], the alterations observed at S3 may develop as a means of avoiding a worsening of respiratory function.

Our data suggest that, in PCM patients with severe PS, wound healing develops with higher intensity in the S1-S2 period, i.e., between the clinical and the serological cure. The specific causes underlying the dysregulated, and consequently delayed, wound healing observed in patients with severe PS should be explored in further studies.

Our data show an association between severe PS–with intense fibrosis and emphysema–and dysregulation of wound healing, increasing knowledge of fibrogenesis in PCM and identifying new possible targets for drug investigation: unique and upregulated proteins at S0 such as enolase isoforms, hemoglobin’s, transthyretin, angiotensinogen, complement C5, putative uncharacterized protein MYH16, shieldin complex subunit 3, as well as, some protective downregulated proteins at S0 as apolipoprotein A-I and β2-glicoproteína I [62–64, 75, 76], vitamin D binding protein [77–79], haptoglobin and hemopexin [80–83] and retinol-binding protein 4 [65, 84].

Of five unique serum proteins in severe PS patients at S0, angiotensinogen and complement C5 have been showed an important pro-fibrotic role by activation of myofibroblasts cells. Angiotensinogen (AGT) is an precursor in tissue renin-angiotensin system (RAS) that plays an important role in promoting the development of hepatic fibrogenesis [85], renal interstitial fibrosis [86], and idiopathic pulmonary fibrosis [87]. Interesting, it was observed that a direct inhibition of AGT in pro-fibrotic cells could attenuate the progression of hepatic fibrosis in the early stage [85]. In addition, preliminary clinical studies on chronic hepatitis C and non-alcoholic steatohepatitis suggest that RAS blocking agents may have beneficial effects on progression of fibrosis [88, 89]. Therefore, the study of angiotensinogen in the context of chronic PCM should be better investigate. Complement C5 seems to be a druggable mediator of pancreatic fibrosis that directly activates pancreatic stellate cells and whose deletion or inhibition greatly reduces fibrogenesis after pancreatic necrosis [90]. In addition, the pro-fibrotic role of C5 have been observed also in renal [91] and liver fibrosis [92]. Unique 60S ribosomal protein L7-like 1, a putative uncharacterized protein MYH16, and the shieldin complex subunit 3 had never been associated with wound healing process, its role in fibrosis of PCM should be investigate in future studies.

In the same context, the up-regulated proteins in severe PS patients at S0 are also related with higher activity of pro-fibrotic cells and collagen production as enolase isoforms, hemoglobin´s family and transthyretin. The enolase is an enzyme participating of glycolytic functions and its upregulation have been contributing with diverse pathological process including hepatic fibrosis [93]. In addition, drugs blocking enolase activity has already been investigated [94, 95], but needs to be evaluated in context of chronic PCM. Although hemoglobin family and transthyretin are not usually associated with fibrosis process, a recent study demonstrated that transthyretin affects cardiac fibroblasts, contributing to heart fibrosis [96].

Concerning the downregulated proteins in severe PS patients compared with mild/moderate patients at S0, some of them have also been shown to be altered in fibrotic diseases, such as serum β2-glycoprotein I in Chagas disease [97], and hepatitis C [98]. Vitamin D binding protein in chronic obstructive pulmonary disease [99] and, similar to our findings, plasminogen has been found down-regulated in the plasma of patients with IPF [23]. Also, decreased expression of vitamin D binding and apolipoprotein A-I are indicative of liver fibrosis in patients with hepatitis C [100].

As severe PS leads to a compromised respiratory function in PCM patients, and time points immediately before treatment and at clinical cure are key moments at which PS can progress to severe, we suggest that the prognoses for severe PS should be considered as soon as possible and as early as diagnosis of PCM. For this, we have identified 15 proteins that were unique or most highly upregulated at the moment of diagnosis (before treatment, S0) as predictive biomarkers of severe PS development in PCM, as following: 60S ribosomal protein L7-like 1, angiotensinogen, complement C5, putative uncharacterized protein MYH16, shieldin complex subunit 3, hemoglobin subunit beta, hemoglobin subunit gamma-2, hemoglobin subunit delta, hemoglobin subunit epsilon, hemoglobin subunit gamma-1, hemoglobin subunit alpha, alpha-enolase, gamma-enolase, beta-enolase, transthyretin.

The limitations of this study was our inability to validate the proteins identified by the proteomic approach, the study did not quantifyed the extension of sequelae severity. Others challenges of this study were 1) low number of new cases/year in one center of research; 2) low adherence to visiting during the whole long-term of follow-up; 3) frequent co-morbidities that exclude a high number of patients; 4) poor adherence to the antifungal treatment. Indeed, the present study is a result of more than two decades of hard work that evaluated and followed-up the enrolled patients carefully. In addition, the answer to the question why some patients develop more fibrosis than others when faced same infection remains open and should be further investigated. The strengths of our study were the thorough analysis of differentially expressed proteins, the prospective design, including evaluation at the four clinical follow-up stages, and the analytically useful comparison with patients with mild/moderate PS.

We conclude that severe PS as a PCM outcome results from a dysregulation in important stages of wound healing, especially before treatment and at clinical cure. In addition, we identified the 15 most highly upregulated proteins in patients with severe versus mild/moderate PS immediately before treatment as candidates for predictive severe PS biomarker in PCM. Our findings provide new insights into pulmonary fibrogenesis in PCM patients and a guide for further studies on antifibrotic treatments in combination with antifungal therapies.

## Supporting information

S1 Table Proteins with expression significantly altered in the serum of paracoccidioidomycosis patients with severe and mild/moderate pulmonary sequel (PS) as outcome in the moment of before treatment (S0). (DOCX)

S2 Table Proteins with expression significantly altered in the serum of paracoccidioidomycosis patients with severe and mild/moderate pulmonary sequel (PS) as outcome in the moment of clinical cure (S1). (DOCX)

S3 Table Proteins with expression significantly altered in the serum of paracoccidioidomycosis patients with severe and mild/moderate pulmonary sequel (PS) as outcome in the moment of serological cure (S2). (DOCX)

S4 Table Proteins with expression significantly altered in the serum of paracoccidioidomycosis patients with severe and mild/moderate pulmonary sequel (PS) as outcome in the moment of apparent cure (S3). (DOCX)
