# Supplementary material for: Proteomic analysis of serum samples of paracoccidioidomycosis patients with severe pulmonary sequel
Source: PLoS Negl Trop Dis. 2021 Aug 23;15(8):e0009714. doi: 10.1371/journal.pntd.0009714 (PMC8425554; doi:10.1371/journal.pntd.0009714)
Supplement: S1 Table — (DOCX) [file pntd.0009714.s001.docx]

| **S1 Table**. Proteins with expression significantly altered in the serum of paracoccidioidomycosis patients with severe and mild/moderate pulmonary sequel (PS) as outcome in the moment of before treatment (S0). | | | |
| --- | --- | --- | --- |
| **^a^Access number** | **Protein name** | **PLGS Score** | **^b^*Ratio* (severe PS:mild/moderate PS)** |
| P68871 | Hemoglobin subunit beta | 5774 | 2,34 |
| P69892 | Hemoglobin subunit gamma-2 | 2216 | 2,32 |
| P02042 | Hemoglobin subunit delta | 2216 | 2,29 |
| P02100 | Hemoglobin subunit epsilon | 2216 | 2,27 |
| P69891 | Hemoglobin subunit gamma-1 | 2216 | 2,25 |
| P69905 | Hemoglobin subunit alpha | 4673 | 2,16 |
| P06733 | Alpha-enolase | 556 | 1,30 |
| P09104 | Gamma-enolase | 556 | 1,30 |
| P13929 | Beta-enolase | 556 | 1,30 |
| P02766 | Transthyretin | 3869 | 1,17 |
| P02750 | Leucine-rich alpha-2-glycoprotein | 124 | 0,36 |
| P01860 | Immunoglobulin heavy constant gamma 3 | 2528 | 0,38 |
| P01857 | Immunoglobulin heavy constant gamma 1 | 8472 | 0,41 |
| P00739 | Haptoglobin-related protein | 8443 | 0,43 |
| P00738 | Haptoglobin | 19050 | 0,43 |
| P01859 | Immunoglobulin heavy constant gamma 2 | 1115 | 0,44 |
| P01023 | Alpha-2-macroglobulin | 4747 | 0,44 |
| P20742 | Pregnancy zone protein | 196 | 0,47 |
| P0DOY3 | Immunoglobulin lambda constant 3 | 9212 | 0,48 |
| P0CG04 | Immunoglobulin lambda constant 1 | 8860 | 0,48 |
| P0DOY2 | Immunoglobulin lambda constant 2 | 9212 | 0,50 |
| P01011 | Alpha-1-antichymotrypsin | 1432 | 0,50 |
| P02749 | Beta-2-glycoprotein 1 | 847 | 0,51 |
| P01009 | Alpha-1-antitrypsin | 3334 | 0,51 |
| P0C0L4 | Complement C4-A | 316 | 0,51 |
| P01619 | Immunoglobulin kappa variable 3-20 | 353 | 0,51 |
| P0C0L5 | Complement C4-B | 316 | 0,52 |
| P02760 | Protein AMBP | 607 | 0,53 |
| P04433 | Immunoglobulin kappa variable 3-11 | 204 | 0,57 |
| P01834 | Immunoglobulin kappa constant | 6687 | 0,58 |
| P04217 | Alpha-1B-glycoprotein | 995 | 0,58 |
| A0A0A0MRZ8 | Immunoglobulin kappa variable 3D-11 | 204 | 0,59 |
| P01024 | Complement C3 | 9892 | 0,59 |
| P02790 | Hemopexin | 3711 | 0,60 |
| P00747 | Plasminogen | 238 | 0,60 |
| P00450 | Ceruloplasmin | 603 | 0,61 |
| P00751 | Complement factor B | 1014 | 0,61 |
| Q5T013 | Putative hydroxypyruvate isomerase | 163 | 0,63 |
| P02774 | Vitamin D-binding protein | 1196 | 0,64 |
| P01861 | Immunoglobulin heavy constant gamma 4 | 2315 | 0,65 |
| P0DP02 | Immunoglobulin heavy variable 3-30-3 | 431 | 0,65 |
| P0DP03 | Immunoglobulin heavy variable 3-30-5 | 431 | 0,65 |
| P08603 | Complement factor H | 299 | 0,66 |
| P01767 | Immunoglobulin heavy variable 3-53 | 431 | 0,66 |
| A0A0B4J1X5 | Immunoglobulin heavy variable 3-74 | 431 | 0,66 |
| P01768 | Immunoglobulin heavy variable 3-30 | 431 | 0,66 |
| P01764 | Immunoglobulin heavy variable 3-23 | 431 | 0,67 |
| P01772 | Immunoglobulin heavy variable 3-33 | 431 | 0,67 |
| A0A0C4DH42 | Immunoglobulin heavy variable 3-66 | 431 | 0,67 |
| A0M8Q6 | Immunoglobulin lambda constant 7 | 6622 | 0,67 |
| P04004 | Vitronectin | 455 | 0,67 |
| P05546 | Heparin cofactor 2 | 96 | 0,68 |
| B9A064 | Immunoglobulin lambda-like polypeptide 5 | 8860 | 0,71 |
| P10909 | Clusterin | 762 | 0,72 |
| P0CF74 | Immunoglobulin lambda constant 6 | 9212 | 0,72 |
| P19827 | Inter-alpha-trypsin inhibitor heavy chain H1 | 228 | 0,72 |
| P19823 | Inter-alpha-trypsin inhibitor heavy chain H2 | 769 | 0,76 |
| Q14624 | Inter-alpha-trypsin inhibitor heavy chain H4 | 94 | 0,79 |
| P01871 | Immunoglobulin heavy constant um | 668 | 0,79 |
| P01042 | Kininogen-1 | 436 | 0,80 |
| P00734 | Prothrombin | 362 | 0,80 |
| P02787 | Serotransferrin | 52105 | 0,82 |
| P02647 | Apolipoprotein A-I | 5802 | 0,87 |
| P02765 | Alpha-2-HS-glycoprotein | 1470 | 0,88 |
| P01877 | Immunoglobulin heavy constant alpha 2 | 3884 | 0,89 |
| P01876 | Immunoglobulin heavy constant alpha 1 | 6654 | 0,90 |
| Q6DKI1 | 60S ribosomal protein L7-like 1 | 34 | Severe PS* |
| P01019 | Angiotensinogen | 109 | Severe PS |
| P01031 | Complement C5 | 32 | Severe PS |
| Q9H6N6 | Putative uncharacterized protein MYH16 | 49 | Severe PS |
| Q6ZNX1 | Shieldin complex subunit 3 | 36 | Severe PS |
| P02763 | Alpha-1-acid glycoprotein 1 | 493 | mild/moderate PS |
| P19652 | Alpha-1-acid glycoprotein 2 | 1020 | mild/moderate PS |
| Q02985 | Complement factor H-related protein 3 | 20 | mild/moderate PS |
| Q5SZK8 | FRAS1-related extracellular matrix protein 2 | 12 | mild/moderate PS |
| P01762 | Immunoglobulin heavy variable 3-11 | 430 | mild/moderate PS |
| P01766 | Immunoglobulin heavy variable 3-13 | 430 | mild/moderate PS |
| A0A0C4DH32 | Immunoglobulin heavy variable 3-20 | 430 | mild/moderate PS |
| A0A0B4J1V1 | Immunoglobulin heavy variable 3-21 | 430 | mild/moderate PS |
| A0A0B4J1X8 | Immunoglobulin heavy variable 3-43 | 430 | mild/moderate PS |
| P0DP04 | Immunoglobulin heavy variable 3-43D | 430 | mild/moderate PS |
| P01763 | Immunoglobulin heavy variable 3-48 | 430 | mild/moderate PS |
| P01780 | Immunoglobulin heavy variable 3-7 | 430 | mild/moderate PS |
| P01782 | Immunoglobulin heavy variable 3-9 | 430 | mild/moderate PS |
| P01624 | Immunoglobulin kappa variable 3-15 | 578 | mild/moderate PS |
| A0A0C4DH55 | Immunoglobulin kappa variable 3D-7 | 578 | mild/moderate PS |
| P17301 | Integrin alpha-2 | 37 | mild/moderate PS |
| O60229 | Kalirin | 37 | mild/moderate PS |
| Q86W24 | NACHT_ LRR and PYD domains-containing protein 14 | 16 | mild/moderate PS |
| P05155 | Plasma protease C1 inhibitor | 192 | mild/moderate PS |
| O94827 | Pleckstrin homology domain-containing family G member 5 | 72 | mild/moderate PS |
| Q9NZ71 | Regulator of telomere elongation helicase 1 | 70 | mild/moderate PS |
| P02753 | Retinol-binding protein 4 | 104 | mild/moderate PS |
| **^a^** Identification is based on proteins ID from UniProt protein database, reviewed only (http://www.uniprot.org). | | | |
| **^b^** Proteins with expression significantly altered are organizaed according to the ratio. | | | |
| ***** Indicates unique proteins in alphabetical order. | | | |
