# Supplementary material for: Proteomic analysis of serum samples of paracoccidioidomycosis patients with severe pulmonary sequel
Source: PLoS Negl Trop Dis. 2021 Aug 23;15(8):e0009714. doi: 10.1371/journal.pntd.0009714 (PMC8425554; doi:10.1371/journal.pntd.0009714)
Supplement: S2 Table — (DOCX) [file pntd.0009714.s002.docx]

| **S2 Table**. Proteins with expression significantly altered in the serum of paracoccidioidomycosis patients with severe and mild/moderate pulmonary sequel (PS) as outcome in the moment of clinical cure (S1). | | | |
| --- | --- | --- | --- |
| **^a^Access number** | **Protein name** | **PLGS Score** | **^b^*Ratio* (severe PS:mild/moderate PS)** |
| P69905 | Hemoglobin subunit alpha | 2896 | 1,72 |
| A0M8Q6 | Immunoglobulin lambda constant 7 | 3425 | 1,38 |
| P0DOY3 | Immunoglobulin lambda constant 3 | 11696 | 1,34 |
| P0CG04 | Immunoglobulin lambda constant 1 | 11393 | 1,32 |
| P0DOY2 | Immunoglobulin lambda constant 2 | 11696 | 1,32 |
| P0CF74 | Immunoglobulin lambda constant 6 | 8139 | 1,31 |
| B9A064 | Immunoglobulin lambda-like polypeptide 5 | 11393 | 1,31 |
| P02787 | Serotransferrin | 44194 | 1,30 |
| P01877 | Immunoglobulin heavy constant alpha 2 | 4519 | 1,26 |
| P01834 | Immunoglobulin kappa constant | 11313 | 1,23 |
| P01876 | Immunoglobulin heavy constant alpha 1 | 7529 | 1,22 |
| P00738 | Haptoglobin | 21516 | 1,16 |
| P01764 | Immunoglobulin heavy variable 3-23 | 1564 | 1,16 |
| P00739 | Haptoglobin-related protein | 10216 | 1,14 |
| P68871 | Hemoglobin subunit beta | 4951 | 1,11 |
| P01871 | Immunoglobulin heavy constant mu | 2666 | 0,94 |
| P04217 | Alpha-1B-glycoprotein | 948 | 0,93 |
| P01011 | Alpha-1-antichymotrypsin | 2832 | 0,90 |
| P08603 | Complement factor H | 284 | 0,88 |
| P19827 | Inter-alpha-trypsin inhibitor heavy chain H1 | 328 | 0,87 |
| P02774 | Vitamin D-binding protein | 2264 | 0,87 |
| P02751 | Fibronectin | 94 | 0,86 |
| P00747 | Plasminogen | 308 | 0,85 |
| P02749 | Beta-2-glycoprotein 1 | 905 | 0,84 |
| P0C0L4 | Complement C4-A | 470 | 0,84 |
| P0C0L5 | Complement C4-B | 470 | 0,84 |
| P05546 | Heparin cofactor 2 | 94 | 0,84 |
| P04004 | Vitronectin | 763 | 0,82 |
| P01024 | Complement C3 | 11714 | 0,81 |
| P19823 | Inter-alpha-trypsin inhibitor heavy chain H2 | 868 | 0,79 |
| P01023 | Alpha-2-macroglobulin | 8579 | 0,79 |
| P20742 | Pregnancy zone protein | 378 | 0,79 |
| P00450 | Ceruloplasmin | 1877 | 0,78 |
| P01042 | Kininogen-1 | 320 | 0,77 |
| P02760 | Protein AMBP | 590 | 0,76 |
| P01008 | Antithrombin-III | 145 | 0,75 |
| P02790 | Hemopexin | 4428 | 0,72 |
| P01009 | Alpha-1-antitrypsin | 5216 | 0,70 |
| P00734 | Prothrombin | 501 | 0,70 |
| P10909 | Clusterin | 697 | 0,69 |
| P06727 | Apolipoprotein A-IV | 131 | 0,65 |
| P02766 | Transthyretin | 1202 | 0,62 |
| P02647 | Apolipoprotein A-I | 4009 | 0,58 |
| P02656 | Apolipoprotein C-III | 1698 | 0,36 |
| P02763 | Alpha-1-acid glycoprotein 1 | 157 | Severe PS* |
| P01019 | Angiotensinogen | 272 | Mild/moderate PS |
| P02649 | Apolipoprotein E | 130 | Mild/moderate PS |
| P02753 | Retinol-binding protein 4 | 437 | Mild/moderate PS |
| P27169 | Serum paraoxonase/arylesterase 1 | 1266 | Mild/moderate PS |
| **^a^** Identification is based on proteins ID from UniProt protein database, reviewed only (http://www.uniprot.org). | | | |
| **^b^** Proteins with expression significantly altered are organizaed according to the ratio. | | | |
| ***** Indicates unique proteins in alphabetical order. | | | |
