# Supplementary material for: Proteomic analysis of serum samples of paracoccidioidomycosis patients with severe pulmonary sequel
Source: PLoS Negl Trop Dis. 2021 Aug 23;15(8):e0009714. doi: 10.1371/journal.pntd.0009714 (PMC8425554; doi:10.1371/journal.pntd.0009714)
Supplement: S3 Table — (DOCX) [file pntd.0009714.s003.docx]

| **S3 Table**. Proteins with expression significantly altered in the serum of paracoccidioidomycosis patients with severe and mild/moderate pulmonary sequel (PS) as outcome in the moment of serological cure (S2). | | | |
| --- | --- | --- | --- |
| **^a^Access number** | **Protein name** | **PLGS Score** | **^b^*Ratio* (severe PS:mild/moderate PS)** |
| P02751 | Fibronectin | 799 | 2,32 |
| P05155 | Plasma protease C1 inhibitor | 561 | 1,82 |
| P00739 | Haptoglobin-related protein | 7979 | 1,39 |
| P00738 | Haptoglobin | 20464 | 1,35 |
| Q14624 | Inter-alpha-trypsin inhibitor heavy chain H4 | 56 | 1,34 |
| Q5T013 | Putative hydroxypyruvate isomerase | 140 | 1,32 |
| P02787 | Serotransferrin | 58693 | 1,25 |
| P02765 | Alpha-2-HS-glycoprotein | 1410 | 1,22 |
| P02750 | Leucine-rich alpha-2-glycoprotein | 280 | 1,21 |
| P13929 | Beta-enolase | 1050 | 1,19 |
| P09104 | Gamma-enolase | 1050 | 1,19 |
| P01024 | Complement C3 | 21361 | 1,16 |
| P00751 | Complement factor B | 1405 | 1,15 |
| P02790 | Hemopexin | 5861 | 1,15 |
| P04004 | Vitronectin | 693 | 1,15 |
| P04196 | Histidine-rich glycoprotein | 110 | 1,15 |
| P05546 | Heparin cofactor 2 | 158 | 1,12 |
| P00747 | Plasminogen | 465 | 1,09 |
| P01011 | Alpha-1-antichymotrypsin | 2056 | 1,08 |
| P02647 | Apolipoprotein A-I | 8940 | 1,07 |
| P10909 | Clusterin | 1717 | 1,07 |
| P20742 | Pregnancy zone protein | 301 | 1,06 |
| P02774 | Vitamin D-binding protein | 2536 | 1,05 |
| P00450 | Ceruloplasmin | 1149 | 1,04 |
| P01023 | Alpha-2-macroglobulin | 9840 | 1,03 |
| P01834 | Immunoglobulin kappa constant | 6201 | 0,94 |
| P01877 | Immunoglobulin heavy constant alpha 2 | 3499 | 0,93 |
| P06727 | Apolipoprotein A-IV | 126 | 0,91 |
| P0C0L4 | Complement C4-A | 470 | 0,91 |
| P0C0L5 | Complement C4-B | 460 | 0,90 |
| P02760 | Protein AMBP | 336 | 0,89 |
| P01009 | Alpha-1-antitrypsin | 6126 | 0,84 |
| P01876 | Immunoglobulin heavy constant alpha 1 | 7516 | 0,79 |
| P02749 | Beta-2-glycoprotein 1 | 1341 | 0,78 |
| P0DOY2 | Immunoglobulin lambda constant 2 | 10532 | 0,70 |
| P0DOY3 | Immunoglobulin lambda constant 3 | 10532 | 0,70 |
| B9A064 | Immunoglobulin lambda-like polypeptide 5 | 8591 | 0,70 |
| P0CG04 | Immunoglobulin lambda constant 1 | 8591 | 0,70 |
| P0CF74 | Immunoglobulin lambda constant 6 | 6815 | 0,69 |
| A0M8Q6 | Immunoglobulin lambda constant 7 | 4453 | 0,66 |
| P02763 | Alpha-1-acid glycoprotein 1 | 225 | 0,61 |
| P27169 | Serum paraoxonase/arylesterase 1 | 726 | 0,57 |
| P69905 | Hemoglobin subunit alpha | 386 | 0,52 |
| P19652 | Alpha-1-acid glycoprotein 2 | 123 | 0,51 |
| P68871 | Hemoglobin subunit beta | 2416 | 0,50 |
| P02042 | Hemoglobin subunit delta | 1332 | 0,47 |
| P02100 | Hemoglobin subunit epsilon | 1332 | 0,46 |
| P69891 | Hemoglobin subunit gamma-1 | 1332 | 0,46 |
| P69892 | Hemoglobin subunit gamma-2 | 1332 | 0,45 |
| P08697 | Alpha-2-antiplasmin | 689 | Severe PS* |
| P02652 | Apolipoprotein A-II | 436 | Severe PS |
| P02649 | Apolipoprotein E | 158 | Severe PS |
| P0DJI8 | Serum amyloid A-1 protein | 3449 | Severe PS |
| P0DJI9 | Serum amyloid A-2 protein | 544 | Severe PS |
| Q15166 | Serum paraoxonase/lactonase 3 | 4 | mild/moderate PS |
| **^a^** Identification is based on proteins ID from UniProt protein database, reviewed only (http://www.uniprot.org). | | | |
| **^b^** Proteins with expression significantly altered are organizaed according to the ratio. | | | |
| ***** Indicates unique proteins in alphabetical order. | | | |
