# Supplementary material for: Proteomic analysis of serum samples of paracoccidioidomycosis patients with severe pulmonary sequel
Source: PLoS Negl Trop Dis. 2021 Aug 23;15(8):e0009714. doi: 10.1371/journal.pntd.0009714 (PMC8425554; doi:10.1371/journal.pntd.0009714)
Supplement: S4 Table — (DOCX) [file pntd.0009714.s004.docx]

| **S4 Table**. Proteins with expression significantly altered in the serum of paracoccidioidomycosis patients with severe and mild/moderate pulmonary sequel (PS) as outcome in the moment of apparent cure (S3). | | | |
| --- | --- | --- | --- |
| **^a^Access number** | **Protein name** | **PLGS Score** | **^b^*Ratio* (severe PS:mild/moderate PS)** |
| P69905 | Hemoglobin subunit alpha | 1823 | 1,30 |
| P02790 | Hemopexin | 6961 | 1,06 |
| A0M8Q6 | Immunoglobulin lambda constant 7 | 3133 | 0,90 |
| P01871 | Immunoglobulin heavy constant mu | 2221 | 0,89 |
| P08603 | Complement factor H | 486 | 0,88 |
| P00738 | Haptoglobin | 22790 | 0,87 |
| P0CG04 | Immunoglobulin lambda constant 1 | 6325 | 0,87 |
| P01023 | Alpha-2-macroglobulin | 6037 | 0,85 |
| P0DOY2 | Immunoglobulin lambda constant 2 | 6524 | 0,85 |
| P0CF74 | Immunoglobulin lambda constant 6 | 6524 | 0,85 |
| B9A064 | Immunoglobulin lambda-like polypeptide 5 | 6325 | 0,85 |
| P00739 | Haptoglobin-related protein | 9541 | 0,84 |
| P20742 | Pregnancy zone protein | 432 | 0,84 |
| P0DOY3 | Immunoglobulin lambda constant 3 | 6524 | 0,84 |
| P00751 | Complement factor B | 1196 | 0,83 |
| P02749 | Beta-2-glycoprotein 1 | 1413 | 0,78 |
| P01876 | Immunoglobulin heavy constant alpha 1 | 7491 | 0,77 |
| P01834 | Immunoglobulin kappa constant | 2808 | 0,77 |
| P02774 | Vitamin D-binding protein | 1889 | 0,77 |
| P01877 | Immunoglobulin heavy constant alpha 2 | 3640 | 0,76 |
| P00747 | Plasminogen | 337 | 0,76 |
| P04004 | Vitronectin | 705 | 0,76 |
| Q96PD5 | N-acetylmuramoyl-L-alanine amidase | 144 | 0,74 |
| P01042 | Kininogen-1 | 732 | 0,74 |
| P0C0L4 | Complement C4-A | 489 | 0,73 |
| P0C0L5 | Complement C4-B | 488 | 0,73 |
| P00734 | Prothrombin | 541 | 0,73 |
| P05546 | Heparin cofactor 2 | 93 | 0,72 |
| P02766 | Transthyretin | 2160 | 0,72 |
| P01011 | Alpha-1-antichymotrypsin | 1925 | 0,71 |
| P04217 | Alpha-1B-glycoprotein | 999 | 0,68 |
| P00450 | Ceruloplasmin | 2315 | 0,66 |
| P01024 | Complement C3 | 13144 | 0,66 |
| P19827 | Inter-alpha-trypsin inhibitor heavy chain H1 | 1036 | 0,66 |
| P01019 | Angiotensinogen | 117 | 0,65 |
| P02760 | Protein AMBP | 495 | 0,65 |
| P02787 | Serotransferrin | 45136 | 0,65 |
| Q14624 | Inter-alpha-trypsin inhibitor heavy chain H4 | 147 | 0,64 |
| P19823 | Inter-alpha-trypsin inhibitor heavy chain H2 | 1325 | 0,63 |
| Q5T013 | Putative hydroxypyruvate isomerase | 72 | 0,60 |
| P02656 | Apolipoprotein C-III | 4030 | 0,59 |
| P02765 | Alpha-2-HS-glycoprotein | 2620 | 0,58 |
| P10909 | Clusterin | 823 | 0,58 |
| P06727 | Apolipoprotein A-IV | 155 | 0,56 |
| P01009 | Alpha-1-antitrypsin | 4246 | 0,55 |
| P02647 | Apolipoprotein A-I | 8083 | 0,52 |
| P01008 | Antithrombin-III | 156 | Severe PS* |
| P01764 | Immunoglobulin heavy variable 3-23 | 369 | Severe PS |
| P01768 | Immunoglobulin heavy variable 3-30 | 369 | Severe PS |
| P0DP02 | Immunoglobulin heavy variable 3-30-3 | 369 | Severe PS |
| P0DP03 | Immunoglobulin heavy variable 3-30-5 | 369 | Severe PS |
| P01772 | Immunoglobulin heavy variable 3-33 | 369 | Severe PS |
| P01767 | Immunoglobulin heavy variable 3-53 | 369 | Severe PS |
| A0A0C4DH42 | Immunoglobulin heavy variable 3-66 | 369 | Severe PS |
| A0A0B4J1X5 | Immunoglobulin heavy variable 3-74 | 369 | Severe PS |
| P43652 | Afamin | 120 | Mild/moderate PS |
| P02763 | Alpha-1-acid glycoprotein 1 | 279 | Mild/moderate PS |
| P02652 | Apolipoprotein A-II | 547 | Mild/moderate PS |
| P02649 | Apolipoprotein E | 486 | Mild/moderate PS |
| P09871 | Complement C1s subcomponent | 65 | Mild/moderate PS |
| P05155 | Plasma protease C1 inhibitor | 90 | Mild/moderate PS |
| P27169 | Serum paraoxonase/arylesterase 1 | 857 | Mild/moderate PS |
| **^a^** Identification is based on proteins ID from UniProt protein database, reviewed only (http://www.uniprot.org). | | | |
| **^b^** Proteins with expression significantly altered are organizaed according to the ratio. | | | |
| ***** Indicates unique proteins in alphabetical order. | | | |
